# Needs, Challenges and Countermeasures of SARS-CoV-2 Surveillance in Cold-Chain Foods and Packaging to Prevent Possible COVID-19 Resurgence: A Perspective from Advanced Detections

**DOI:** 10.3390/v15010120

**Published:** 2022-12-30

**Authors:** Yaru Li, Jiali Qiao, Xiao Han, Zhiying Zhao, Jun Kou, Wenlu Zhang, Shuli Man, Long Ma

**Affiliations:** 1 State Key Laboratory of Food Nutrition and Safety, Tianjin 300457, China; 2 Key Laboratory of Industrial Microbiology, Ministry of Education, Tianjin 300457, China; 3 Tianjin Key Laboratory of Industry Microbiology, National and Local United Engineering Lab of Metabolic Control Fermentation Technology, Tianjin 300457, China; 4 China International Science and Technology Cooperation Base of Food Nutrition/Safety and Medicinal Chemistry, Tianjin 300457, China; 5 College of Biotechnology, Tianjin University of Science & Technology, Tianjin 300457, China

**Keywords:** SARS-CoV-2, cold-chain foods and packaging, detection methods, COVID-19 resurgence, challenges and countermeasures

## Abstract

The pandemic caused by SARS-CoV-2 has a huge impact on the global economy. SARS-CoV-2 could possibly and potentially be transmitted to humans through cold-chain foods and packaging (namely good-to-human), although it mainly depends on a human-to-human route. It is imperative to develop countermeasures to cope with the spread of viruses and fulfil effective surveillance of cold-chain foods and packaging. This review outlined SARS-CoV-2-related cold-chain food incidents and current methods for detecting SARS-CoV-2. Then the needs, challenges and practicable countermeasures for SARS-CoV-2 detection, specifically for cold-chain foods and packaging, were underlined. In fact, currently established detection methods for SARS-CoV-2 are mostly used for humans; thus, these may not be ideally applied to cold-chain foods directly. Therefore, it creates a need to develop novel methods and low-cost, automatic, mini-sized devices specifically for cold-chain foods and packaging. The review intended to draw people’s attention to the possible spread of SARS-CoV-2 with cold-chain foods and proposed perspectives for futuristic cold-chain foods monitoring during the pandemic.

## 1. Introduction

Infectious diseases caused by viruses have long been considered an important factor that endangers human health and causes social panic. In December 2019, unexplained lungs with fever, fatigue, cough and poor breathing as the main symptoms appeared one after another in Wuhan, Hubei province, China [1]. Less than 2 months later, the World Health Organization (WHO) confirmed and declared COVID-19 on 11 February 2020 [2]. At that time, the Coronavirus Study Group (CSG) of the International Committee on Virus Taxonomy officially named it SARS-CoV-2, in line with a phylogenetic analysis of related coronaviruses [3]. SARS-CoV-2 is spreading and infecting people all over the world and has caused a global pandemic. To date (December 2022), there have been more than 560 million confirmed cases and more than 6.3 million deaths [4].

**Figure 1:**
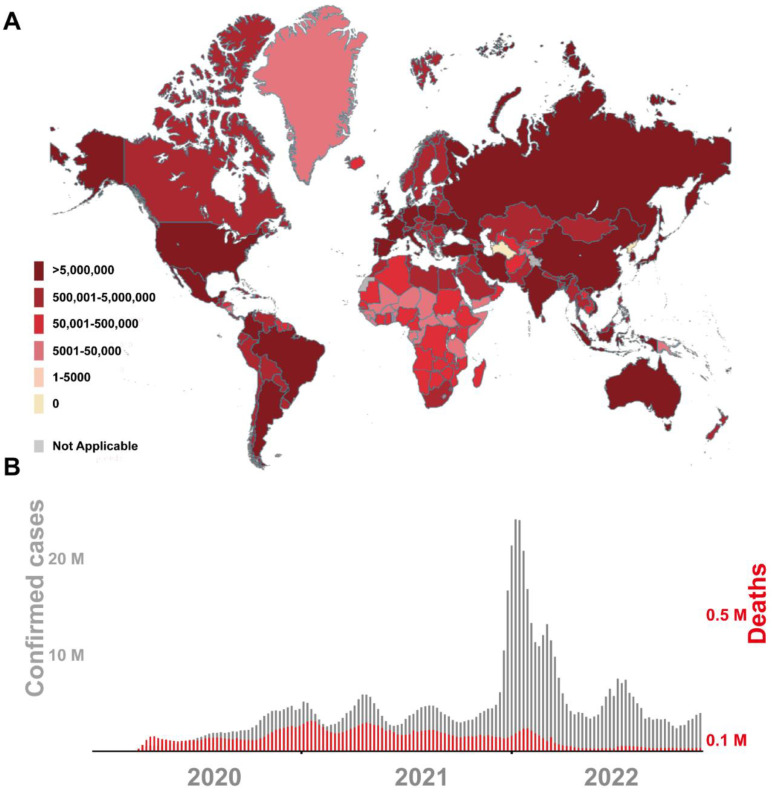
Current global SARS-CoV-2 epidemic situation map (**A**) and number of confirmed cases and deaths (**B**). The figure was obtained on the official website of the World Health Organization (WHO) on 11 November 2020 and 25 November 2020.

Figure 1 demonstrates the current infection situation of SARS-CoV-2 in the world and summarizes the confirmed number of cases and deaths per week since the COVID-19 epidemic. The high rate of infection and death of COVID-19 has taken a huge toll on people’s daily living standard and global economic development. The COVID-19 pandemic has created a new situation we hardly faced before, while we are still in the middle of figuring out the consequences in different aspects [2,5]. Moreover, it is confirmed that exposure to packaging contaminated by SARS-CoV-2 can lead to infection. This has suggested that the SARS-CoV-2 contaminated packaging could be potential hidden dangers and threats [6]. Researchers engaged in food hygiene and safety, public health and preventive medicine, biosensor/bioanalytical method development, virus detection and food safety are actively studying the management and research of SARS-CoV-2, including the detection method of SARS-CoV-2 and its spread in cold-chain foods and packaging [7]. It is extremely imperative to research and develop innovative SARS-CoV-2 diagnostic methods, which are not only limited to humans, but also include items such as the cold-chain foods, outer packaging and environmental samples.

Although the role of contaminated cold-chain foods and packaging materials in transmission of SARS-CoV-2 remains highly controversial and somewhat speculative, cold-chain foods and packaging is a possible and potential hazard for SARS-CoV-2 to spread. Although there is not substantial evidence so far to confirm the transmission of COVID-19 through cold-chain foods, they may serve as a potential carrier to facilitate the spread of SARS-CoV-2. The following things may cause the SARS-CoV-2 transmission: touch with contaminated surfaces and the subsequent touching of the nose, mouth and eye mucosal membranes [8]. Thus, it is not negligible that the possibility of the SARS-CoV-2 spreads from the infected patients to the surface of cold-chain foods [9]. Furthermore, it is proactive to attract attention to the fact that cold-chain foods and packaging contamination is an important driver in the propagation of the SARS-CoV-2 pandemic, and concerns about the cold chain should justify a global emergency to improve detection and traceability.

## 2. Transmission of SARS-CoV-2 in Cold-Chain Foods

**Figure 2:**
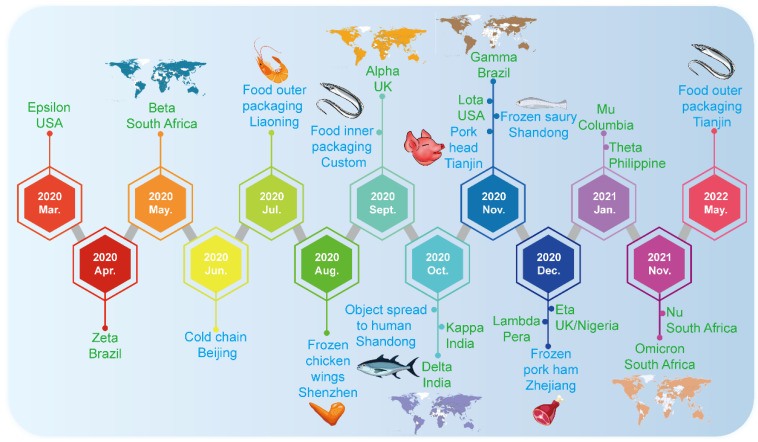
Timeline of the emergence of SARS-CoV-2 variants and SARS-CoV-2-related cold-chain food incidents. The blue font indicates the time point when novel coronavirus was detected in cold-chain foods in China. The green font indicates the timeline of the emergence of SARS-CoV-2 variants and those of greater concern. The worldwide spread of five variants of concern (VOCs) is also shown.

The safety of cold-chain foods does not comprise an exception and it has become a new challenge [7]. Cold-chain logistics is used for temperature-sensitive food, etc., which usually requires the foods to be refrigerated (2–8 °C) or frozen (<−18 °C) in the whole process [10]. In June 2020, COVID-19 broke out in the Xinfadi market, Beijing’s largest wholesale food market [11]. The Beijing Center for Disease Prevention and Control (CDC) found a great similarity between the SARS-CoV-2 in imported frozen salmon and the strain in the human cases from the market. They speculated that the source of the epidemic was likely to be initiated from contaminated imported salmon [12]. The spread of SARS-CoV-2 carried by imported cold-chain foods immediately aroused widespread concern [13]. Furthermore, it was found that a low temperature was a suitable and congenial environment for the survival of SARS-CoV-2, which provided appropriate opportunities and conditions for long-distance transmission [14,15]. Since then, local COVID-19 outbreaks related to cold-chain foods have occurred in Kashgar, Tianjin, Shanghai, Dalian and other places as well (Figure 2) [16]. All index cases in these events had the experience of contacting and handling imported frozen food or its containers before diagnosis; however, epidemiological investigation indicated that there was no intersection or contact between these cases and SARS-CoV-2 positive people. Subsequently, SARS-CoV-2 was tested many times in cold-chain foods; in particular, for imported cold-chain foods, such as imported frozen white shrimp, purchased Chilean cherries, imported pitaya, etc [17]. Notably, it is worth mentioning that the CDC and prevention detected and isolated live viruses from the packaging of cold-chain foods carried by workers during the traceability investigation of COVID-19 in Qingdao, in September 2020, for the first time [13]. Epidemiological information showed that the two workers were infected after contacting contaminated outer packaging. It has been persuasively proven that cold-chain products and packaging can act as the carrier of SARS-CoV-2, leading to local transmission in the ports, cold storage or seafood markets, and even community outbreaks. However, it has been reported that the decontamination of SARS-CoV-2 from cold-chain food packaging has no marginal benefit in risk reduction for food workers through the quantitative microbial risk assessment model using frozen food packaging facilities [18]. There are several reviews which summarized the characteristics, risks and preventive countermeasures of SARS-CoV-2 transmission [19]. Furthermore, they emphasized that the international community should pay close attention to the SARS-CoV-2 cold-chain transmission; in addition, the disinfection of cold-chain products and the personnel protective measures in production, processing, transportation and sale of cold-chain products should also be strengthened to prevent the silent transmission or reintroduction of SARS-CoV-2 by cold chain.

Up to now, transmission and infection caused by SARS-CoV-2 in cold-chain foods were reported and brought serious consequences. The event of a SARS-CoV-2 outbreak caused by cold-chain foods is exhibited in Figure 2. More cold-chain foods have been tested as COVID-19-positive samples in different parts of China. The potential risk of SARS-CoV-2 spreading through cold-chain foods and the emerging challenge to cold-chain foods safety is a major issue.

Furthermore, Figure 2 illustrates the timeline of the emergence of SARS-CoV-2 variants. Since the discovery of the novel coronavirus, with the continuous adaptation of the virus to environmental changes, a variety of mutant strains have emerged. In order to avoid stigmatizing the countries concerned, WHO named the mutant strain after the Greek alphabet. So far, 14 mutant strains have been named; five of them are listed as variants of concern (VOC), Alpha, Beta, Gamma, Delta and Omicron [20]. The first variant, Alpha, was first discovered in the UK, Beta was first discovered in South Africa, Gamma was first detected in Brazil, Delta was first discovered in India in October 2020 [21] and the latest variant, Omicron, was first reported in South Africa in November 2021, and quickly concerned WHO [22]. Delta and Omicron strains are the most infectious variant strains among them, and they are the main variants prevalent in the world at present.

## 3. The Current Status of Detection Methods for SARS-CoV-2

**Figure 3:**
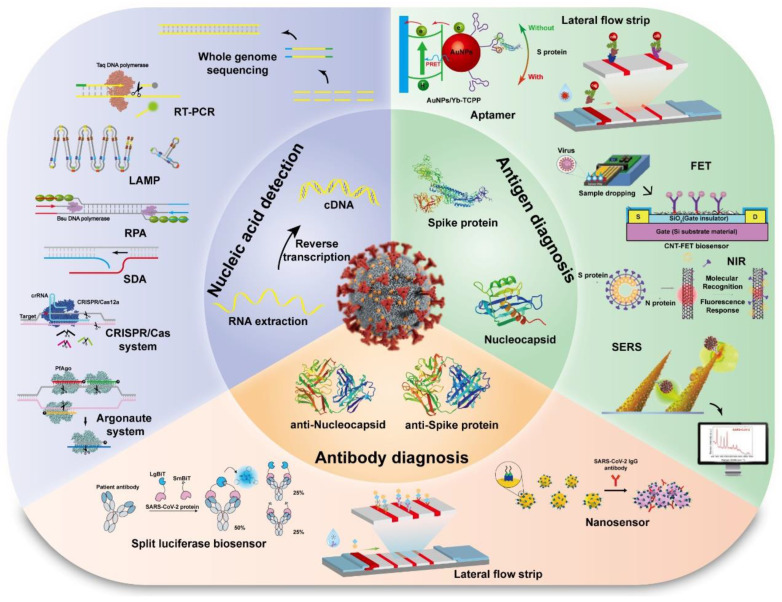
The detection methods for SARS-CoV-2 based on the biological-recognition elements.

It has been proven that prompt and accurate monitoring is an effective and optimal settlement for the reduction and management of virus transmission and epidemic track. The traditional diagnostic method for epidemic virus infection is to culture, isolate and identify the virus, which takes a few days and is inferior and low in sensitivity and specificity compared with nucleic acid detection. The method is helpful for identifying pathogens at an early stage. However, large-scale screening is not applicable because of time-consuming, tediousness and low efficiency [14]. With this method, it is easy to cause virus pollution, transmission and infection in the meantime. We outlined and reviewed the development for detecting SARS-CoV-2 according to their respective biological-recognition elements, such as nucleic acid, antigen, antibody and other pathogen markers as shown in Figure 3.

### 3.1. Molecular Detection

Molecular detection technology is the most direct and necessary assay and measure for food pathogen pollution. RT-PCR is currently the gold standard for monitoring SARS-CoV-2 molecular diagnostic methods [23]. Trace amounts of viral genes in the sample mixture can be effectively amplified by RT-PCR [24]. However, this measurement is a multi-step technique that relies on enzymes, complex machines and trained operational professionals, which may be incapable and not suitable for large-scale rapid and timely screening, such as in the food industry and cold-chain foods safety monitoring, to some extent. Many researchers turn to isothermal techniques and portable real-time devices [25]. Their properties, independence of thermal cycling instruments, isothermal performance and point-of-need make it a great possibility to integrate these technologies into cold-chain transportation for on-site setting diagnostics, creating a cost-effective and time-efficient approach. Isothermal amplification method is performed at a constant temperature by using various enzymes and strategies without the requirement of expensive equipment, such as thermal circulators [26], including rolling circle amplification (RCA), loop-mediated isothermal amplification (LAMP), recombinase polymerase amplification (RPA), etc. Chaibun et al. combined RCA with electrochemistry to establish an ultra-sensitive biosensor with rapidity for detecting SARS-CoV-2 [27]. The one-step sandwich hybridization could obtain a detection limit as low as 1 copy /μL of N and S genes within 2 h. Bokelmann et al. achieved an accurate and low-cost detection of SARS-CoV-2 by improving LAMP, combining RNA extraction from hybridization capture and color scoring of smartphones [28]. The results obtained from sample collection took less than 1 h, and the method was applicable and compatible to POCT. Furthermore, the research described a strategy for sequence-specific detection of SARS-CoV-2 genome, which improved discrimination by adding molecular beacons to the simple reaction mixture coupled with RT-LAMP [29]. This method offered a reasonable and simple SARS-CoV-2 RNA detection method, which was available for screening large-scale populations. Moreover, a variety of nanomaterials were presented and applied to detect SARS-CoV-2 with capable and appropriate performance, particularly in the food industry and cold-chain foods [30]. Li et al. fabricated a gold nanoparticle-decorated graphene field-effect transistor sensor to quickly identify the SARS-CoV-2 virus using clinical throat swab samples [31].

The strategy of next-generation sequencing plays an important role in the tracking and dissemination of SARS-CoV-2 and helps to establish effective and rapid molecular diagnostic methods [32]. The researchers established the SARS-CoV-2 genomes sequencing platform and built a repository and database of SARS-CoV-2 genome data and related metadata, which could provide good data support for solving the problems caused by SARS-CoV-2 [33]. A platform was proposed for parallel SARS-CoV-2 detection on tens of thousands of samples for saliva analysis. A toehold switch was introduced as a bio-selective recognition element to design an inductance capacitance (LC) resonator sensor, which was employed to detect the SARS-CoV-2 virus specifically [34]. The toe switch would be relaxed because of the presence of SARS-CoV-2 N gene, and then protease could be expressed to degrade the gelatin switch and alter the resonance frequency of the planar resonance sensor.

### 3.2. CRISPR/Cas and Argonaute Based Detection

In addition, guided, programmable and target-activated nucleases, exemplified by CRISPR/Cas system and Argonaute, are emerging as a new generation of nucleic acid [35,36,37,38,39]. Detection methods based on CRISPR [40,41] and Argonaute [42,43] have also been developed and applied thoroughly in order to establish portable and rapid diagnostic assays for SARS-CoV-2 to fulfill the requirements in different scenarios. The CRISPR/Cas system has attracted interest because of its high-precision gene editing capability and trans-cutting activity, providing a more specific, convenient and reliable method for food safety field testing [44,45,46]. CRISPR/Cas9-mediated LFA, assisted with RT-RPA, was developed to detect simultaneous dual-gene SARS-CoV-2 in a single strip test with rapidity and sensitivity [38]. An analytical method based on CRISPR/Cas12a for ultra-sensitive detection of SARS-CoV-2 was proposed in one pot with visualization [47]. A contamination-free visual detection method with LAMP and CRISPR/Cas12a technology was developed for SARS-CoV-2. The tube lid was used to pre-add CRISPR/Cas12a reagents. After LAMP reaction, CRISPR/Cas12a reagents flowed into the tube and mixed with amplicon solution with hand shaking. The result can be seen with the naked eye, without any dedicated instruments, with the help of smart phones and portable 3D printing instruments. The whole time was 40 min with high sensitivity [48]. A smartphone readout-based biosensor was devised for ultrasensitive and selective detection of SARS-CoV-2, powered by CRISPR/Cas12a. The dis-aggregation of gold nanoparticles could generate observable color changes. It rendered “single copy resolution”, as evidenced by the 1 copy/μL limit of detection of pseudoviruses, with no cross-reactivity, and presented 100% agreement with qPCR results [40]. By using suboptimal protospacer adjacent motifs for producing stronger fluorescence, a one-step fluorescence assay was developed to detect SARS-CoV-2 RNA in nasopharyngeal samples within 20 min [49]. CRISPR/Cas12a was combined with a commercial pregnancy test strip (PTS) to translate the target to colorimetric signal on the PTS with a cell phone app and a hand-held microchip quantification [37]. Cas13a was also applied in SARS-CoV-2 detection [50]. Graphene field-effect transistors were introduced to eliminate the pre-amplification. A novel biosensor based on Cas13a was used to detect SARS-CoV-2 with target amplification free. The assay can detect as low as 1 attomolar with ultra-sensitivity [51]. By combining the advantages of mobile phone microscope, Fozouni et al. proposed a non-amplification CRISPR/Cas13a detection method for directly detecting SARS-CoV-2 in RNA of nasal swabs, which realized a sensitivity of approximately 100 copies/μL in 30 min [52]. de Puig et al. reported miSHERLOCK based on CRISPR for highly sensitive multiplex detection of SARS-CoV-2 and its related mutants with low cost and POC tests [53]. In addition to the above-mentioned detection methods, there are also diagnostic platforms based on Cas13d [54], engineered AsCas12a [55], etc. Technologies such as microfluidics [56], nanopore sensing strategy [57], gold nanoparticles [58] and so on are used to establish a more comprehensive and applicable SARS-CoV-2 detection for various needs.

Additionally, Argonaute-mediated biosensors could become the next generation nucleic acid detection platforms [59]. Argonaute is a nucleic acid-oriented endonuclease and, unlike CRISPR/Cas, prefers to be guided by short 5′-phosphorylated single-stranded DNA, and cleaves DNA substrates without the presence of proto-spatial adjacent motifs (PAM). There is no specific sequence restriction on the selection of target DNA cleavage sites. Wang et al. redefined *Pyrococcus furiosus* (*Pf*Ago)- based nucleic acid detection and developed a highly sensitive and precise molecular diagnostic strategy to identify SARS-CoV-2 and mutants, owing to a specific single nucleotide. It took approximately 1 h in the whole experiment [42]. Xun et al. reported a *Pf*Ago-based detection platform for detecting SARS-CoV-2 accurately and sensitively with speed, scalability and portability [60]. Furthermore, another study has achieved multiple detection by integrating isothermal amplification technology with Argonaute, which can detect multiplex targets of SARS-CoV-2 at the same time [43]. Furthermore, other nucleases have been employed and applied in virus detection.

### 3.3. Virus Protein Detection/Antigen Detection

In addition to nucleic acid test for early diagnosis, a virus can be quantified or qualitied by immunoassay [61]. Specific antibodies, high-performance enzyme labelling and robust analytical techniques are important factors for detecting stably and sensitively. Antigen diagnosis is the main immunological method of food quarantine. Unlike PCR-based approaches, antigen tests do not require thermal amplification steps and directly detect viral components, like spike glycoprotein, envelope protein, etc. Antigen immunoassay is performed by the high selectivity of antigen-antibody reaction to identify the virus, which only needs to mix the sample with the designed probe. A portable infrared spectrometer with a purpose-built transfection accessory was applied to a rapid screening and testing for SARS-CoV-2 [62]. The field-effect transistor was also used to construct a biosensor for the detection of SARS-CoV-2 spike protein by coating a specific antibody on graphene [63]. This method demonstrated a higher sensitivity in culture medium and clinical samples. In addition, to achieve sensitive point-of-care methods, magnetic quantum dots were introduced to establish a dual-mode lateral flow immunoassay biosensor, which could be used to simultaneously and ultra-sensitively detect SARS-CoV-2 spikes and nucleocapsid protein antigens [64]. A non-reagent virus sensing was proposed and applied to directly analyze SARS-CoV-2 and its relevant spike proteins within 5 min, which only used a sensor-modified electrode chip and was based on the dynamic and kinetic response of the probe and virus compound [65].

Based on the high infectivity and pathogenicity of SARS-CoV-2, there is also a certain risk for direct immunoassay of the virus. Immunoassay based on antibody could make up the current detection methods due to the characteristics of simplicity, sensitivity and rapidity in clinical diagnosis.

### 3.4. Other Detections

Virus determination would be achieved through designing specific antibodies to recognize the virus [61]. On this basis, the medical community has widely used antibody testing for the clinical diagnosis of viruses. However, since antibodies cannot be produced in cold-chain foods or packaging, there are no reports suggesting testing until now. Therefore, the antibody test is presented here to offer guidance and references for the future research and detection of SARS-CoV-2 in the food field, and support researchers looking to exploit new applications or diagnostic techniques for SARS-CoV-2 monitoring, especially in cold-chain foods.

Commonly used rapid detection methods, such as enzyme-linked immunosorbent assays, lateral flow assays or chemiluminescent microparticle immunoassays, can be a reference for carrying out screening of the COVID-19 epidemic. Immunoassay can make up or balance the problems of workload, false negative results and aerosol pollution based on nucleic acid detection methods. Elledge et al. developed a fast, affordable solution-based assay to detect antibodies for SARS-CoV-2 using ingeniously designed split luciferase antibody biosensors, which greatly reduced the complexity and increased scalability of SARS-CoV-2 detection [66]. In addition, Lew et al. reported an analysis platform to detect SARS-CoV-2 antibodies with cost-effectiveness and time-effectiveness, based on the interactions between specific epitope and IgG [67]. SARS-CoV-2 antibodies and epitope-functionalized AuNPs would bind through bivalent specificity, which resulted in aggregated nanoparticles, and the AuNPs plasmonic features exhibited distinct optical transitions within 30 min. The detection limit of this method is 3.2 nM, which is comparable to IgG levels in patients recovering from COVID-19. Compared with nucleic acid tests, samples for antibody testing are more readily available, greatly reducing the risk of infection for health care workers during collection and testing and facilitating screening in primary laboratories. Some researchers have developed and reported a strategy with effectiveness and timeliness to produce high-affinity antibodies or antibody-like proteins, based on SARS-CoV-2 spike protein and other detection objects [68]. The monomer sequence against the SARS-CoV-2 spike protein could be successfully obtained in only 4 days, and then it was applied to catch SARS-CoV-2 particles from patients’ nasal swab samples. Zhao et al. proposed a protein biosensor with electronic labelling strategy in combination with colloidal quantum dot modified electrode for rapid, accurate and convenient analysis of SARS-CoV-2 [69]. In addition, there are other potential alternative methods, such as other biomarkers for SARS-CoV-2, the construction of colorimetry or the combination of the aforementioned methods.

The RNA-directed and de novo RNA replicable function of RdRP was introduced to develop a novel diagnostic methodology for COVID-19, based on an RNA platform for RdRP-induced transcription [70]. Aptamer was also used to develop an aptasensor combined with a screen-printed carbon electrode platform, and the receptor-binding domain in the spike protein of the SARS-CoV-2. The aptasensor can yield a limit of detection of 1.30 pM (66 pg/mL) [71]. Fluidic-atomic force microscopy mediated nanomechanical deflection was reported to specifically monitor SARS-CoV-2 antigenic proteins in just a few minutes, based on a microcantilever-based optical detection [72].

The detection of SARS-CoV-2 mutant strains is extremely vital, due to the enhancement of the infectivity and transmission of mutations [73]. CRISPR/Cas12a-based detection provided an optimum solution for the monitoring and diagnosis of Omicron because of its specific recognition [74]. Zhong et al. integrated primer-encoded microsphere technology and dual fluorescence decoding strategy to establish an encodable, multiple microsphere phase amplification sensing platform, which could identify 10 key single-nucleotide variants in the receptor-binding region simultaneously for the detection of SARS-CoV-2 variants of concern [75]. Furthermore, an electrochemical nano biosensor system was proposed to detect SARS-CoV-2 RNA and its mutant derivatives, without labelling and amplification, which was combined with 3D printing technology to achieve multi-channel detection [76]. A light-up CRISPR/Cas13 method was used to detect SARS-CoV-2 and its mutated variants with the sensitivity as low as 82 copies [77].

Furthermore, various technologies were applied for SARS-CoV-2 detection, such as surface-enhanced Raman scattering [78], droplet magneto-fluidic device [79], surface plasmon resonance [80] and so on.

## 4. Challenges and Countermeasures

Currently established detection methods for SARS-CoV-2 are mostly used for humans (patients, personnel under quarantine, etc.), and the majority are not applicable or appropriate to be directly applied on cold-chain foods. Due to the particularity of cold-chain foods, such as the influence and interaction of the cold-chain food matrix, low abundance and uneven distribution of the virus on the outer package in cold-chain foods, the necessity of timely on-site inspection, higher requirements and stricter standards and principles are put forward for sampling methods, detection performance, anti-interference ability and field detection capability.

### 4.1. The Sample Pretreatment

Most methods for detecting SARS-CoV-2 in cold-chain foods are nucleic acid and antigen assays. The sample pretreatment is inevitable and necessary prior to the detection. Diverse foods make a distinction between composition, such as protein substances (fish), carbohydrates and plant fibers (fruits) and so on. Studies have shown that SARS-CoV-2 has a longer survival time in protein foods. The seafoods of a cold chain become a potential possibility. The extraction method of nucleic acid or virus would change with the variety or composition of the matrix. The interaction between substrates is also pivotal and urgent to overcome. Furthermore, the distribution of the virus is uneven on the cold-chain foods, and the concentration, as well as the content of the virus, is maintained at a trace level. It is easy to cause sampling errors with the test result, and even “false negative” results happen with adopting swabs sampling method. Pre-amplification is necessary, but bias in nucleic acid amplification and the cross contamination of samples has also been introduced. There are the same troubles or problems in cold-chain food packaging. At present, relative detection methods have also been established to minimize or refrain from the impact of sample pretreatment and food matrixes. Some studies have proposed that the frozen water used for cold-chain foods melted; soaking fluid in cold-chain packaging, etc., could be applied as the media or candidate related to the detection of virus in cold-chain foods [15]. Zhao et al. proposed an integrated platform for directly detecting SARS-CoV-2, which was called an electrochemical system, integrating reconfigurable enzyme-DNA nanostructures. Target induced molecular activation is seamlessly converted into enhanced electrochemical signals, leveraging responsive molecular nanostructures and automated microfluidics [81]. Zhang et al. introduced surface enhanced Raman scattering into the detection of SARS-CoV-2 without any sample pretreatment. SERS immunoassay was two layers of dense and uniform gold nanoparticle films fabricating a novel oil/water/oil (O/W/O), three-phase liquid-liquid interfaces self-assembly method [82]. Similarly, a novel ultra-sensitive surface enhanced Raman spectroscopy sensor drove with enzyme free signal amplification was presented for detecting SARS-CoV-2 RNA directly with rapidity and reliability, which was coupled with SERS-active silver nanorods sensing chips and specially designed smart unlocking mediated target cycle signal amplification strategy [83]. Some researchers have introduced a polyethyleneimine-assisted copper in situ growth strategy to achieve signal amplification to improve the detection performance of gold nanoparticle-based lateral flow sensors with sensitivity [84].

### 4.2. Infectivity of Viruses

The amount of virus is positively correlated with its infectivity within a certain range, because SARS-CoV-2 in cold-chain foods is mainly transmitted by close contact with people. There is a positive correlation between the number of viruses and the probability of contacting with a human. If the viruses could be quantitatively analyzed and detected, it would help to judge the potential hazards and infections of its transmission. Most strikingly, viruses in cold-chain foods have the difference between infectivity and non-infectivity [85]. If this phenomenon can be preliminarily distinguished, it would be helpful for the preliminary hazard assessment of cold-chain foods contaminated by SARS-CoV-2. Therefore, it is necessary to quickly identify and dispose of relevant SARS-CoV-2 pollution events and minimize the extent of its diffusion, owing to the strong diffusion and liquidity of cold-chain foods. The detection method based on photosensitive dyes-propidium monoazide (PMA) has emerged as an analytical tool for the quantitative detection of virus infectivity [86]. In light of the accuracy of the detection and safety of cold-chain foods, there is an increasing need for establishing the real-time detection techniques monitoring different aspects with high sensitivity and point of need [87,88]. The lateral flow biosensor (LFB) based on nanoparticles is a good component and tool for field and portable detection. Zhu et al. combined mRT-LAMP with LFB for detecting SARS-CoV-2, introducing labelled FITC, digoxin and biotin by amplification simultaneously. Signals and results were interpreted through immune reaction with multiple on-site analysis [89].

### 4.3. Quantitative PCR Detection Method

Quantitative PCR is the mainstream molecular technology for SARS-CoV-2 detection [6]. On the one hand, while the detection limit is still not low enough, it is easy to form a “gray area” for detection, resulting in missed and suspicious detection. On the other hand, expensive instruments are necessary and indispensable for the detection of collected samples. The collected nucleic acids are likely to be degraded during the storage and transportation of the collected samples, leading to “false negative” results. At the same time, the risk of infection and transmission also increase. The BC (blockchain) and IoT (Internet of Things) could provide a possibility and potential in ensuring the quality and safety of cold-chain foods. A supply chain architecture supporting BC was proposed to guarantee the availability of tamper proof audit trails to deal with COVID-19 in the frozen meat supply chain [90]. Qian et al. conjointly analyzed COVID-19 and food cold-chain systems, and developed an improved conceptual system for cold-chain food management in combination with the BC and IoT, using the results of the analysis [87]. In addition, an intelligent dynamic prediction model based on knowledge rules was constructed, which could integrate a flexible humidity sensor into the non-destructive monitoring of the IoT to provide real-time feedback and dynamic adjustment for the chilled chicken cold chain [91]. A real-time temperature measurement protocol framework supported by passive RFID, IoT and statistical process control charts was reported to cope with temperature fluctuations and abuse in the cold-chain foods, leading to ensuring the quality of foods [92]. “Beijing cold chain” based on the BC and IoT was launched on 1 November 2020 in Beijing, China, which could realize the electronic traceability management of imported refrigerated frozen meat and aquatic products, and the data docking with the national cold-chain foods traceability platform. Furthermore, a traceability system based on RFID technology and IoT services has been designed and carried out in a real scenario to track cold-chain food conditions [93]. It is reported that the IoT and artificial intelligence technologies (such as machine learning and diagnostic deep learning) can be combined with the SARS-CoV-2 diagnostic method, based on POC sensing technology, to investigate useful information through data storage, sharing and analysis [94]. BC and IoT could be a helpful or possible approach to combat SARS-CoV-2 transmission from cold-chain foods.

In addition, the detection schemes based on antigen antibody reaction and aptamer are still in their infancy, which is still a certain distance from real application. A one-step thermophoretic assay was reported to directly quantify and detect SARS-CoV-2 viral particles utilizing polyethylene glycol and an aptamer binding to the spike protein [95]. Furthermore, a haemagglutination test was developed to detect antibodies to the receptor binding domain of the SARS-CoV-2 spike protein with point-of-care-test and no requirement for special equipment. Zhou et al. devised a non-enzymatic virus detection strategy by mechanically reconfiguring for reacting to specific viruses with DNA nanoswitches [96].

All the above facts and inferences urgently require the establishment of a novel detection strategy for SARS-CoV-2 in cold-chain foods. The purpose is to find problems in time, take measures quickly, send the tested positive samples to professional laboratories and improve turn-around time for further inspection, when necessary, to give full play to the characteristics of rapid detection and make full use of laboratory resources to form an all-round monitoring system.

### 4.4. Safety Precautions in Cold-Chain Foods

**Figure 4:**
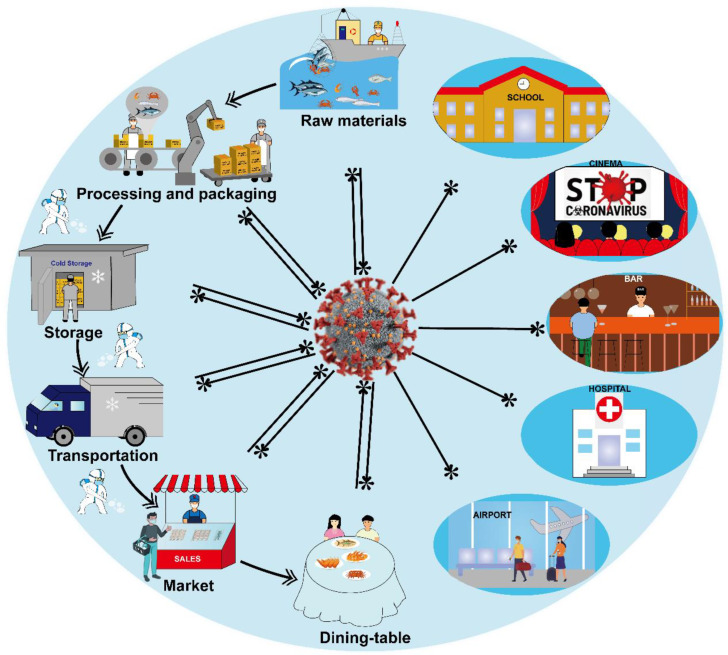
The potential transmission and infection of SARS-CoV-2 in cold-chain links, such as foods, personnel involved in the operation, the whole cold-chain links, waste and disinfection and sterilization procedures, etc.

Figure 4 presents the key control points of cold-chain foods closely interlinked to SARS-CoV-2 pollution, from raw materials to table consumption [97]. Most strikingly, it is of great importance to monitor the safety of cold-chain foods every step of the way, from production to delivery in real-time with traceability, the data of the personnel engaged in the cold-chain operation, disinfection and sterilization for cold-chain foods, which results in enhancing protection, traceability and safety within the cold-chain supply. The application of BC and IoT is an effective guarantee and means for the detection of cold-chain foods and monitoring the presence of viruses in a timely manner, during the transmission of cold-chain foods, to control the virus infection and avoid large-scale infection. The traceability and timely detection of the whole cold-chain process should be taken into account [98].

For cold-chain foods, the process to the dining table consists of raw materials, the processing, packaging and storage, the transportation of processed food, the sales of products, and the final consumers’ consumption, which holds a low ideal temperature range to guarantee the quality of cold-chain foods. Unfortunately, SARS-CoV-2 can survive under low temperature conditions, and long-distance propagation provides ideal opportunities for SARS-CoV-2. Cold-chain foods contain a high viral concentration or prolonged exposure to contaminated food, leading to a risk of SARS-CoV-2 transmission. Thus, the potential safety risks and precaution measures during the cold-chain links should be timely monitored and sequentially analyzed.

For the personnel engaged in the cold-chain operation, first, the same as cold-chain foods, establish relevant databases for them, including daily health requirements, registration and on duty health registration forms. In addition, disinfection and sterilization work for the personnel should be done thoroughly and comprehensively to prevent the potential virus from being brought back to homes and spreading to other safe places. Last but not least, the personnel should perform virus detection before or after work, and the detection data should be operated in the same way as cold-chain foods to make sure all kinds of information is searchable [87].

Research has indicated that the live virus can be detected within 72 h after being applied to plastic and stainless steel and is more stable on these surfaces compared to copper and cardboard [99,100]. Food packages are generally plastic, which are carefully packed and completely disinfected with the recommended chemicals, including the container [18]. Furthermore, during the storage and transportation of cold-chain foods, it is necessary to carefully inspect the incoming goods and adopt large-scale and effective virus disinfection and sterilization procedures. It is also highly important to further detect and supervise the safety of cold-chain foods after processing, and containers for food packaging are difficult to thoroughly clean up.

In addition, the generation of waste is also inevitable in whole cold-chain foods [101]. On the basis of the type of waste, it needs to be collected, recycled and properly isolated to minimize the risk of infection transmission spread through aerosols and the possibility of virus transmission route (contamination spreading to cold-chain foods and humans) [102]. Simultaneously, the virus load in the waste was monitored regularly and there was an attempt to implement measures to monitor infections.

It is necessary and crucial to detect and monitor SARS-CoV-2 during the cold-chain links to prevent and refrain problems in a certain link quickly and accurately. More importantly, the improvement and enhancement of risk prediction and the safety guarantee of cold-chain foods are imperative and should not be ignored because of the strong infectivity, radiation and harmfulness of SARS-CoV-2 [87]. There are some advances and improvements in SARS-CoV-2 detection methods at present, which could be combined with designed engineering equipment with low-cost, automatic, mini-sized features (recognition of temperature, humidity, gas, etc.), specifically for cold-chain food in future research.

## 5. Concluding Remarks

Cold-chain foods have proved to be a possible way for SARS-CoV-2 to spread, which is not optimistic. The concern about the safety of cold-chain foods, including foods, personnel involved in the operation, the whole cold-chain links, waste and disinfection and sterilization procedures, is still not enough and limited. In short, a suitable and satisfactory detection with high sensitivity, high specificity and on-site capability is still a great necessity in the real supply chain to stop the spread of the virus through efficient screening measures. Warning, tracking and detection at an early stage should be integrated for mitigating and monitoring SARS-CoV-2. In principle, monitoring on site is ideal and optimal, such as performing at home, in a supermarket or any place where samples are provided. Smart and disposable biosensors are required as terminal detection modules with basic network technologies. In summary, we intended to draw people’s attention to the possible spread of COVID-19 within cold-chain foods and proposed the perspective to devise a smart solution for futuristic cold-chain foods monitoring in every individual step from production, delivery and consumption during the pandemic.

## Data Availability

The data presented in this study are available on request from the corresponding author.
